# ProGRP与NSE对小细胞肺癌诊断价值的*meta*分析

**DOI:** 10.3779/j.issn.1009-3419.2010.12.03

**Published:** 2010-12-20

**Authors:** 纪文 王, 佳 高, 捷 赫

**Affiliations:** 100021 北京，北京协和医学院中国医学科学院肿瘤医院肿瘤研究所 Peking Union Medical College, Cancer Hospital, Chinese Academy of Medical Sciences, Beijing 100021, China

**Keywords:** 胃泌素释放肽前体, 神经元特异性烯醇化酶, 肺肿瘤, *meta*分析, Pro-gastrin-releasing peptide, Neuron specifc enolase, Lung neoplasms, meta-analysis

## Abstract

**背景与目的:**

胃泌素释放肽前体（pro-gastrin-releasing peptide, ProGRP）和神经元特异性烯醇化酶（neuron specific enolase, NSE）是目前研究较多的小细胞肺癌（small cell lung cancer, SCLC）肿瘤标记物，本研究综合评价了二者对SCLC的诊断价值。

**方法:**

检索Pubmed、OVID、Elsevier Sciencedirect、Springer、Cochrane Library、Em-base、IFCC、中国生物医学文献数据库和维普医药信息资源系统，收集血清ProGRP与NSE用于SCLC诊断的研究数据。通过*meta*分析拟合SROC曲线，合并诊断效应量，比较ProGRP和NSE对SCLC的诊断效能。

**结果:**

本次*meta*分析共纳入10篇文献，累计病例2 536例，其中SCLC 935例，非小细胞肺癌（non-small cell lung cancer, NSCLC）1 601例，对照849例。ProGRP和NSE的合并敏感度分别为0.70和0.61，合并特异度为0.93和0.90，合并阳性似然比为11.57和5.67，合并阴性似然比为0.32和0.45，合并诊断比值比为36.45和13.08。SROC曲线显示，ProGRP和NSE的Q^*^统计量分别为0.804 2和0.723 2，但差异无统计学意义。

**结论:**

ProGRP对SCLC的鉴别能力与NSE相近，但特异度更高，可以作为SCLC的诊断指标。

## Abstract (en)

**Background and objective:**

Pro-gastrin-releasing peptide (ProGRP) and neuron specific enolase (NSE) have become hotspot of tumor markers for small cell lung cancer (SCLC), and the aim of this study is to evaluate and compare the diagnostic value of serum ProGRP and NSE in SCLC by *meta*-analysis.

**Methods:**

All the English and Chinese literatures for differential diagnosis of SCLC by serum ProGRP and NSE were collected. Sensitivity, specificity, likelihood ratio and diag- nostic odds ratio were pooled using random-effect model. Summary receiver operating characteristic curve analysis was used to evaluate the diagnostic value of serum ProGRP and NSE.

**Results:**

Ten studies were included in the *meta*-analysis, with a total of 2 536 cases. The sensitivity, specificity, positive likelihood ratio, negative likelihood ratio and diagnostic odds ratio of ProGRP in the diagnosis of SCLC were 0.70, 0.93, 11.57, 0.32 and 36.45, and as for NSE those were 0.61, 0.90, 5.67, 0.45 and 13.08, respectively. The Q^*^ value of ProGRP and NSE were 0.804 2 and 0.723 2. There was no statistical significance between the two.

**Conclusion:**

Comparing with NSE, ProGRP has higher specificity and similar discrimination ability, so ProGRP might be a better index in the diagnosis of SCLC.

2007年全球肺癌新发病例约150万，占年新发肿瘤的12%；因肺癌死亡的病例数占年癌症致死数的17.6%，是癌症的首位死因^[1]^。小细胞肺癌（small cell lung cancer, SCLC）约占肺癌总数的20%-25%，其生物学特性和临床特点异于其它病理类型的肺癌。SCLC恶性程度较高，对化疗敏感，其治疗原则与非小细胞肺癌（non-small cell lung cancer, NSCLC）有较大差异。因此寻找能用于SCLC诊断及鉴别诊断的分子标志物具有重要意义。

神经元特异性烯醇化酶（neuron specific enolase, NSE）和胃泌素释放肽前体（pro-gastrin-releasing peptide, ProGRP）均是SCLC相关的血清学诊断标志物。NSE已在临床应用多年，是目前SCLC首选的实验室诊断指标，它在SCLC中的阳性率为60%-81%，但诊断的特异性有待提高，7%-42%的NSCLC和11%-14%的非恶性肿瘤患者，NSE呈假阳性^[2]^。ProGRP是近年来研究较多的具有临床应用前景的肿瘤标志物。研究^[3]^表明，它在鉴别SCLC和其他肺部疾病方面具有较高的敏感度和特异度。

ProGRP对SCLC的诊断价值是否优于NSE，各项研究尚未得到一致结论，亦无大样本量的多中心研究予以证实。本研究通过*meta*分析，综合评价ProGRP与NSE诊断SCLC的价值，以期为ProGRP的进一步研究和临床应用提供参考。

## 1 材料与方法

### 1.1 文献检索

检索Pubmed、OVID、Elsevier Sciencedirect、Springer、Cochrane Library、Embase、IFCC、中国生物医学文献数据库和维普医药信息资源系统，收集2010年5月之前公开发表的关于ProGRP与NSE在肺癌中的血清浓度及其诊断意义的文献。使用的检索词包括neuron specific enolase、pro-gastrin-releasing peptide、NSE、ProGRP、Lung Neoplasms、lung cancer、diagnosis、神经元特异性烯醇化酶、胃泌素释放肽前体、肺癌。检索语言为英语与汉语。

### 1.2 文献纳入与排除标准

文献纳入标准：①研究类型为含有ProGRP和NSE对SCLC诊断价值的前瞻性或回顾性研究；②研究对象涵盖肺癌及肺部良性疾患，采用病理诊断为金标准，文献需明确说明受试者为SCLC、NSCLC或其它病理类型；③文章提供了ProGRP及NSE检测在各病例组的真阳性、真阴性、假阳性、假阴性例数或通过文章提供的数据可以计算；④每组病例数均 > 20。

文献排除标准：①对照组仅有正常人；②ProGRP或NSE的检测方法不是定量检测；③重复性实验中，发表较早或样本量较小的文献排除。

### 1.3 资料提取和质量评价

由2名评价者独立按照预先制定的纳入排除标准筛选文献，根据QUADAS（quality assessment of diagnostic accuracy studies）评价标准^[4]^对纳入文献进行质量评价（总分33分，评分结果见表 1），提取数据并交叉核对，意见不统一时协商解决或参考第三方意见。提取的资料包括文献基本信息、实验设计及实验原始数据（真阳性、假阳性、真阴性及假阴性的例数）。

**1:** 纳入文献的基本特征 General characteristics of included trials

First author	Country	Study type	Blinded design	Consecutive or random	Reference standard	Cases	Quality score
Schneider^[10]^	Germany	Prospective	Unknown	Consecutive	Histology	298	28
Stieber^[11]^	Germany	Retrospective	Unknown	Unknown	Histology	314	27
Molina^[12]^	Spain	Prospective	Unknown	Consecutive	Histology	802	27
Nissan^[13]^	Israel	Prospective	Unknown	Consecutive	Histology	162	28
Shibayama^[14]^	Japan	Unknown	Unknown	Consecutive	Histology	359	26
Lamy^[15]^	France	Retrospective	Yes	Unknown	Histology	245	29
Takada^[16]^	Japan	Retrospective	Yes	Consecutive	Histology	326	30
Yamaguchi^[17]^	Japan	Unknown	Yes	Consecutive	Unknown	602	29
Sun^[18]^	China	Unknown	Unknown	Unknown	Histology	100	27
Yang^[19]^	China	Unknown	Unknown	Unknown	Histology	144	26
Quality score: score by using criteria from the quality assessment of diagnostic accuracy studies (QUADAS).							

### 1.4 统计分析

#### 1.4.1 异质性分析

采用*Q*检验分析纳入研究之间是否存在异质性，以*I*^2^估算分析异质性的大小，然后根据异质性分析的结果选择合适的统计分析模型进行后续的*meta*分析。

#### 1.4.2 *meta*分析

对各研究的原始数据（真阳性、假阳性、真阴性及假阴性的例数）进行整合，采用*DerSimonian Laird*随机效应模型分别计算ProGRP和NSE的平均敏感度、特异度、似然比及各自的95%可信区间（confidence interval, CI）。

采用*Mose’s constant*线性模型拟合SROC曲线^[5]^，以诊断比值比（diagnositic odd ratio, DOR）^[6]^、曲线下面积（aera under curve, AUC）和Q^*^统计量评价诊断试验ProGRP和NSE对SCLC诊断的准确度，并以*Z*检验分析二者诊断的准确性是否存在差异。

计算真阳性率和假阳性率的*Spearman*相关系数*ρ*，分析是否存在阈值效应。以*meta*回归（*REML*法）分别分析ProGRP和NSE异质性的可能来源^[7]^。

以每次减少1篇文献的方法进行敏感性分析，评价本次分析的稳定性^[7]^。以漏斗图和*Egger*线性回归评价纳入的研究是否存在发表偏倚^[8, 9]^。

本文统计用软件为STATA 10和Meta-Disc 1.4，以*P* < 0.05为有统计学差异。

## 2 结果

### 2.1 检索结果及纳入文献

通过设定的检索词进行初步检索，共找到164篇文献。阅读文题和摘要排除143篇，初步纳入文献21篇。进一步阅读全文，排除未达到纳入标准的文献6篇，重复文献3篇，无法获得所需全部原始数据的文献2篇，最终纳入文献共10篇。文献的纳入过程详见图 1。

**1:**
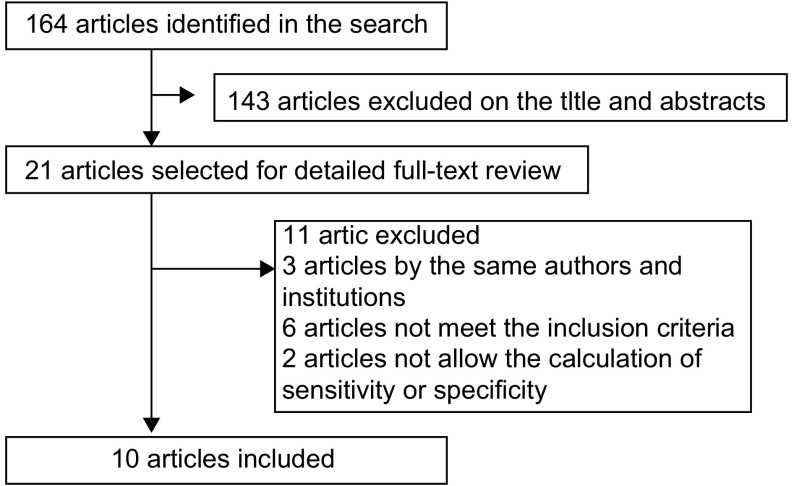
选择文献流程图 Study identification, inclusion and exclusion for *meta*-analysis

### 2.2 纳入研究的基本特征和质量评价

本文共纳入10项研究，累计病例2 536例（SCLC 935例，NSCLC 1 601例），对照849例。各研究的研究设计详见表 1，ProGRP和NSE的检测方法、cutoff值及真阳性、假阳性、假阴性、真阴性的例数参见表 2。

**2:** 纳入研究中ProGRP和NSE检测的实验数据 Summary of results of ProGRP and NSE in included studies

First author	ProGRP							NSE					
	Assay method	Cutoff (pg/mL)	TP	FP	FN	TN		Assay method	Cutoff (ng/mL)	TP	FP	FN	TN
Schneider^[10]^	ELISA	29.1	35	18	16	229		ELISA	9.6	38	35	13	212
Stieber^[11]^	ELISA	38.3	41	9	46	218		RIA	11.9	39	44	48	183
Molina^[12]^	ELISA	50	134	79	41	548		ELISA	25	114	50	61	577
Nissan^[13]^	ELISA	48	29	6	8	119		ELISA	22	18	12	19	113
Shibayama^[14]^	ELISA	49	74	11	40	234		ELISA	7.5	49	10	65	235
Lamy^[15]^	ELISA	53	117	2	29	97		ELISA	17	110	4	36	95
Takada^[16]^	ELISA	33.8	73	22	28	203		ELISA	10.6	63	43	38	182
Yamaguchi^[17]^	ELISA	50	80	6	47	469		ELISA	8.1	79	26	48	449
Sun^[18]^	ELISA	50	25	6	9	60		ECLIA	16.3	19	8	15	58
Yang^[19]^	ELISA	46	46	9	17	72		ECLIA	16.3	40	16	23	65
ELISA: enzyme linked immunosorbent assay; RIA: radioimmunoassay; ECLIA: electro-chemiluminescence immunoassay; TP: true positive; FP: false positive; FN: false negative; TN: true negative; ProGRP: pro-gastrin-releasing peptide; NSE: neuron specific enolase.													

### 2.3 异质性检验

以DOR作为效应量，分别分析ProGRP和NSE的异质性，*Q*检验显示*Cochran-Q*分别为23.93和58.37，*P*均 < 0.05，*I*^2^分别为62.4%和84.6%，研究间均存在异质性，故以下分析均选用随机效应模型。

### 2.4 ProGRP和NSE检测SCLC的敏感度、特异性和似然比

图 2所示为ProGRP和NSE对SCLC诊断敏感度的森林图。ProGRP鉴别SCLC和肺部其他肿物（NSCLC与肺良性疾患）的平均敏感度为0.70（95%CI: 0.67-0.73），NSE的平均敏感度为0.61（95%CI: 0.58-0.64）。图 3所示为ProGRP和NSE对SCLC诊断特异度的森林图。ProGRP鉴别SCLC和肺部其他肿物（NSCLC与肺良性疾患）的平均特异度为0.93（95%CI: 0.92-0.94），NSE的平均特异度为0.90（95%CI: 0.88-0.91）。

**2:**
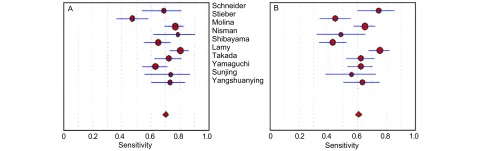
ProGRP（A）和NSE（B）的敏感度森林图 Forest plots of sensitivity of ProGRP (A) and NSE (B)

**3:**
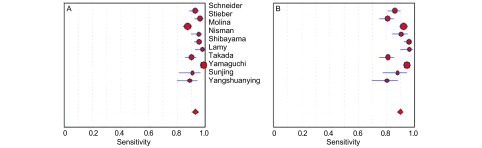
ProGRP（A）和NSE（B）的特异度森林图 Forest plots of specificity of ProGRP (A) and NSE (B)

此外，ProGRP和NSE诊断SCLC的阳性似然比（positive likelihood ratio, PLR）分别为11.57（95%CI: 7.71-17.39）和5.67（95%CI: 3.83-8.39）；阴性似然比（negative likelihood ratio, NLR）分别为0.32（95%CI: 0.26-0.40）和0.45（95%CI: 0.37-0.55），DOR分别为36.45（95%CI:24.12-55.10）和13.08（95%CI: 7.70-22.23），数据详见表 3。

**3:** ProGRP和NSE的合并敏感度、合并特异性、合并似然比 Pooled sensitivity, pooled specificity, and pooled likelihood ration of ProGRP and NSE

	Pooled sensitivity (95%CI)	Pooled specificity (95%CI)	Pooled Positive LR (95%CI)	Pooled Negativee LR (95%CI)	Pooled DOR (95%CI)
ProGRP	0.70 (0.67-0.73)	0.93 (0.92-0.94)	11.57 (7.71-17.39)	0.32 (0.26-0.40)	36.45 (24.12-55.10)
NSE	0.61 (0.58-0.64)	0.90 (0.88-0.91)	5.67 (3.83-8.39)	0.45 (0.37-0.55)	13.08 (7.70-22.23)
LR: likelihood ration; DOR: diagnostic odd ratio; CI: confidence interval.					

### 2.5 ProGRP和NSE鉴别SCLC与其它肺部肿物的SROC曲线

图 4所示为ProGRP和NSE诊断SCLC的SROC曲线。ProGRP和NSE的AUC分别为0.873 7（SE_AUC_=0.039 1）和0.785 4（SE_AUC_=0.915 0），Q^*^统计量分别为0.804 2（SE_Q^*^_=0.027 5）和0.723 2（SE_Q^*^_=0.065 1）。对Q^*^统计量进行*Z*检验（*Z*=1.146, *P* > 0.05），二者没有明显差异。

**4:**
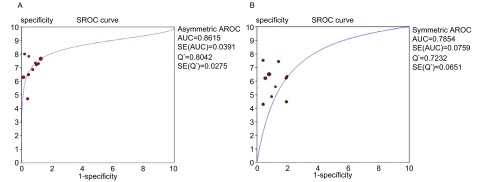
ProGRP（A）和NSE（B）的SROC曲线 SROC curve of ProGRP (A) and NSE (B)

### 2.6 研究间异质性的来源分析

#### 2.6.1 是否存在阈值效应

由ProGRP和NSE的SROC曲线可见，各研究对应的点散在分布，不呈“肩臂”状外观，计算灵敏度对数与（1-特异性）对数的*Spearman*相关系数*ρ*，ProGRP和NSE的*ρ*值分别为0.321和-0.067，*P*均 > 0.05，提示不存在阈值效应。

#### 2.6.2 *meta*回归分析可能的来源

以*meta*回归（*REML*法）分别分析ProGRP和NSE异质性的来源，自变量选择如下：发表年限、样本量、研究设计（前瞻性设计或者回顾性设计）、患者的选择（是否随机）、是否采用盲法、检测方法（ProGRP的检测方法是商品化试剂盒或自建ELISA检测；NSE的检测方法是否是ELISA）。结果详见表 4。由表可见，检测方法是NSE异质性的来源，将其引入*meta*回归分析模型后，方差分量（τ）由0.594 3降为0.299 4，表明检测方法可以解释49.6%的异质性来源。

**4:** *meta*回归分析异质性的来源 Possible sources of heterogeneity of *meta*-analysis

	ProGRP				NSE		
	Coef	*Z*	*P*		Coef	*Z*	*P*
Republic year	-0.042	-0.92	0.355		-0.007	-0.12	0.907
Cases	0.000	0.05	0.963		0.001	1.2	0.229
Study type	-0.107	-0.39	0.700		-0.141	-0.42	0.673
Consecutive or random	0.148	0.31	0.760		0.421	0.76	0.449
Blinded design	0.815	1.80	0.072		0.740	1.29	0.198
Assay method	-0.427	-0.80	0.423		1.163	2.54	0.011
Coef: coefficient.							

### 2.7 敏感度分析

以每次减少1篇文献的方法进行敏感性分析，评估单个研究对本次*meta*分析的影响。表 5显示的是，删除1篇文献后计算的合并DOR及其95%CI。可见，无论剔除哪篇文献，剔除之后的合并DOR均未发生明显变化，提示本次分析结果并未过分依赖于某个研究，结论稳定。

**5:** 各研究对*meta*分析结果的敏感度分析 The influence of each trial for the outcome of the *meta*-analysis

First author	ProGRP			NSE	
	DOR	95%CI		DOR	95%CI
Schneider^[10]^	38.07	24.03-60.30		12.69	7.08-22.69
Stieber^[11]^	39.04	24.89-61.22		15.38	9.82-24.10
Molina^[12]^	39.83	24.98-63.50		12.31	6.83-22.17
Nissan^[13]^	34.39	22.55-52.44		13.62	7.68-24.17
Shibayama^[14]^	36.46	23.02-57.75		12.68	7.09-22.68
Lamy^[15]^	32.57	22.44-47.29		11.24	6.71-18.82
Takada^[16]^	39.08	24.53-62.28		14.15	7.92-25.28
Yamaguchi^[17]^	29.92	21.61-41.41		11.88	6.83-20.68
Sun^[18]^	37.50	24.10-58.35		13.54	7.67-23.93
Yang^[19]^	38.76	24.81-60.55		14.02	7.91-24.84
Combined	36.38	24.17-54.77		13.08	7.7-22.22

### 2.8 发表偏倚

以DOR对数的标准误（SE_LogDOR_）为纵坐标，以Log（DOR）为横坐标，绘制漏斗图，如图 5所示。*Egger*线性回归显示*P* > 0.05，未发现发表偏倚。

**5:**
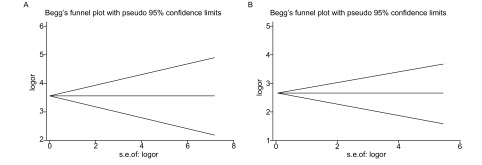
ProGRP（A）和NSE（B）的漏斗图 Funnel graph for ProGRP (A) and NSE (B)

## 3 讨论

本文对纳入的10项研究进行*meta*分析，通过合并诊断效应量、拟合SROC曲线比较ProGRP和NSE对SCLC的诊断效能，通过分析研究间异质性及其来源查找可能影响研究结果的因素，最后通过敏感度分析和检测发表偏倚评估本次*meta*的可信度。

本次评价结果显示，ProGRP和NSE的合并DOR分别为36.45和13.08，提示二者均与SCLC有明显的相关性，且ProGRP的相关性更高。用于鉴别SCLC和NSCLC时，ProGRP的敏感性和特异性均优于NSE，此外，文献资料^[20]^显示ProGRP的假阳性主要来自肾衰患者，排除肾功能异常患者后，其假阳性率仅为2.5%，较好地弥补了NSE特异度不足的缺点。

SROC曲线显示，ProGRP和NSE的AUC分别为0.873 7和0.785 4，Q^*^统计量分别为0.804 2和0.723 2，说明二者鉴别SCLC和其它肺部肿物的准确度均较高。尽管ProGRP的AUC和Q^*^均高于NSE，但*Z*检验无统计学差异，说明ProGRP的鉴别能力并未明显优于NSE。

由于SROC算法复杂，在临床实际工作中应用较少，相比之下，似然比的应用更为简便。一般认为，PLR > 10或NLR < 0.1，基本可以确定或排除诊断。本研究得出的ProGRP和NSE诊断SCLC的PLR分别为11.57和5.67，提示ProGRP阳性可以辅助临床医师做出相应判断，具有更高的临床应用价值。但ProGR P和NSE的NLR分别为0.32和0.45，提示二者阴性时不能排除SCLC的可能。

本文纳入的研究间存在高度异质性。经*Spearman*相关系数检验，异质性与阈值效应无关，因此我们进一步做*meta*回归，试图寻找ProGRP和NSE异质性的可能来源。结果显示，发表年限、样本量、研究类型、患者的选择是否随机、是否采用盲法均不是异质性的来源，说明试验设计的差异不会造成研究结果的明显变化。然后，我们将检测方法作为协变量进行*meta*回归分析。由于纳入的研究ProGRP的检测方法均为ELISA，无法在方法学上进一步分类，因此只以是否采用商品化试剂盒作为亚组分析，结果显示这同样不是ProGRP异质性的来源。NSE已在临床应用多年，其检测方法包括ELISA、电发光、化学发光等，在我们纳入的10项研究中，7项采用ELISA方法检测NSE的浓度，2项研究采用电化学发光法检测，1项采用放射免疫分析法检测，*meta*回归显示检测方法的不同是造成NSE异质性的原因之一，可以解释49.6%的异质性来源。种族或许是异质性的来源，但纳入的文献均未详细报告纳入人群的种族，因此本次研究无法进行分析。

为了判断本研究的稳定性，我们以每次减少1篇文献的方法进行敏感性分析。结果显示，每次减少1篇研究后，合并DOR均未发生明显变化，提示本次分析受单个研究的影响较小，分析结果稳定可信。

本次*meta*分析的局限性：①*meta*分析的局限性：检索到的文献不够全面。检索范围局限在已经发表的研究，对于未公开发表的研究，如会议论文无法获取，可能漏检一些灰色文献；检索语种局限于中文和英文，可能会漏检其它语种的相关研究；②纳入研究的局限性：ProGRP和NSE作为诊断性试验，采用盲法检测和盲法判断可尽量减少诊断的倾向性，而多数研究未报告是否采用盲法检测，存在测量偏倚的可能性。多数研究的病例组均为组织学确诊的病例，然而良性疾患对照组的诊断标准，有些研究并未指明，病例组和对照组诊断标准不一致，可能会导致结果的偏差。

综上所述，ProGRP与NSE有相似的诊断效能，但ProGRP的假阳性率更低，较NSE有一定的优越性，具有临床应用前景。
